# Novel upregulation of amyloid-β precursor protein (APP) by microRNA-346 via targeting of APP mRNA 5′-untranslated region: Implications in Alzheimer’s disease

**DOI:** 10.1038/s41380-018-0266-3

**Published:** 2018-11-23

**Authors:** Justin M. Long, Bryan Maloney, Jack T. Rogers, Debomoy K. Lahiri

**Affiliations:** 1 Department of Psychiatry, Laboratory of Molecular Neurogenetics, Indiana University School of Medicine, Indianapolis, IN 46202 USA; 2 Stark Neuroscience Research Institute, Indiana University School of Medicine, Indianapolis, IN 46202 USA; 3 Neurochemistry Laboratory, Department of Psychiatry-Neuroscience, MGH, Harvard Medical School, Charlestown, MA 02129 USA; 4 Department of Medical and Molecular Genetics, Indiana University School of Medicine, Indianapolis, IN 46202 USA; 5 Indiana Alzheimer Disease Center, Indiana University School of Medicine, Indianapolis, IN 46202 USA

**Keywords:** Neuroscience, Biological techniques, Cell biology, Molecular biology

## Abstract

In addition to the devastating symptoms of dementia, Alzheimer’s disease (AD) is characterized by accumulation of the processing products of the amyloid-β (Aβ) peptide precursor protein (APP). APP’s non-pathogenic functions include regulating intracellular iron (Fe) homeostasis. MicroRNAs are small (~ 20 nucleotides) RNA species that instill specificity to the RNA-induced silencing complex (RISC). In most cases, RISC inhibits mRNA translation through the 3′-untranslated region (UTR) sequence. By contrast, we report a novel activity of miR-346: specifically, that it targets the APP mRNA 5′-UTR to upregulate APP translation and Aβ production. This upregulation is reduced but not eliminated by knockdown of argonaute 2. The target site for miR-346 overlaps with active sites for an iron-responsive element (IRE) and an interleukin-1 (IL-1) acute box element. IREs interact with iron response protein1 (IRP1), an iron-dependent translational repressor. In primary human brain cultures, miR-346 activity required chelation of Fe. In addition, miR-346 levels are altered in late-Braak stage AD. Thus, miR-346 plays a role in upregulation of APP in the CNS and participates in maintaining APP regulation of Fe, which is disrupted in late stages of AD. Further work will be necessary to integrate other metals, and IL-1 into the Fe-miR-346 activity network. We, thus, propose a “FeAR” (Fe, APP, RNA) nexus in the APP 5′-UTR that includes an overlapping miR-346-binding site and the APP IRE. When a “healthy FeAR” exists, activities of miR-346 and IRP/Fe interact to maintain APP homeostasis. Disruption of an element that targets the FeAR nexus would lead to pathogenic disruption of APP translation and protein production.

## Introduction

Alzheimer’s disease (AD) is a neurodegenerative disorder most typical of old age (65 +). The disease is characterized by extracellular neuritic plaques that consist mostly of amyloid-β (Aβ) peptide. Neurofibrillary tangles (NFT) of hyperphosphorylated tau occur within neurons, whereas gliosis, neuroinflammation, and synaptic loss are also evident in the hippocampi and brain cortices of affected individuals [1, 2]. Although autosomally dominant inherited (familial) forms of AD exist, they constitute no more than  5% of AD cases [2]. AD (familial and sporadic) is influenced by multiple genetic and environmental factors, and these factors are considered particularly influential in sporadic AD [3, 4], which requires the study of multiple molecular targets, mechanisms, pathways, and therapeutic strategies [5–8]. Significant evidence supports an Aβ-centric view of AD, e.g., carriers of a protective Aβ precursor protein (APP) polymorphism, APP A673T (Icelandic) [9], have reduced: incidence of AD, of Aβ levels throughout their lives, and of Aβ aggregation. The Aβ peptide is cleaved from the APP by β-secretase (or BACE1) and γ-secretase complex [10].

APP has non-pathogenic functions. Although Aβ accumulation is a typical pathological feature of AD, the instigating disease mechanism is still very poorly understood. How could disruption of APP in the normal brain contribute to neuropathogenesis? A vital physiological role for APP is metal regulation, including ferrohomeostasis [11, 12]. Further, Fe stimulates production of APP protein [13–15]. This is particularly relevant given ample evidence of Fe dyshomeostasis in AD [13, 16]. Notably, non-amyloidogenic processing of APP is enhanced by increasing Fe levels, but only up to a threshold, at which point, additional Fe inhibits non-amyloidogenic APP processing [17]. Monomeric Aβ reduces oxidative stress brought about by metals, in particular, monomeric Aβ inhibits reduction of Fe(III) and prevents lipid peroxidation induced by Fe(II) [18]. Current work in the regulation of APP production by Fe has concentrated on an iron-responsive element (IRE) in the APP mRNA 5′-UTR [14, 15, 19, 20].

We have previously shown the regulatory effects of several microRNA (miRNA) species on AD-associated gene products, including miR-101 and miR-153, which act on the APP 3'-UTR [21, 22], and miR-339-5p on the 3'-UTR of the BACE1 transcript [23]. In this context, miRNAs are a unique class of small (~ 22 nt), non-coding RNA that fine-tune gene expression. In particular, miRNAs appear in complex interactive regulatory networks that govern both normal function and sporadic diseases of the central nervous system [24]. Specific miRNAs may even “co-dispose” toward apparently disparate disorders, such as AD and pulmonary fibrosis [25]. Mature miRNA often binds a protein of the argonaute (AGO) family to form RNA-induced silencing complex (RISC). The miRNA allows RISC to recognize sites of imperfect complementarity on target mRNA transcripts. In essence, a specific miRNA is a “socket” that grants sequence specificity. Most known miRNA target sites are in the 3′-untranslated regions (UTRs) of mRNAs. RISC typically inhibits protein synthesis by repressing translation or destabilizing the transcript. APP [21, 22] and BACE1 [23] are among known miRNA targets in AD.

Our process for evaluating the impact of miRNAs for APP expression began with non-presumptive in silico database comparisons between 5′-UTR and 3′-UTR sequences of genes of interest (e.g., APP and BACE1) vs known miRNA seed sequences [26]. We not only predicted but biologically tested multiple potential miRNA regulators of APP [21–23]; miR-346 was found among the database predictions.

Interestingly, miR-346 may have broad neuropsychiatric influence. An analysis of predicted miRNA:mRNA interactions for schizophrenia-associated gene products revealed that miR-346 contains a higher rate of predicted interactions than expected by chance [27]. Of greater interest, miR-346 expression decreased in the brains of schizophrenic and bipolar patients relative to control patients [27]. Paradoxically, elevated miR-346 has also been reported in the blood of schizophrenia patients, with strong diagnostic utility (AUC 0.713; specificity 90.2%) [28]. The coding sequence for pri-miR-346 is hosted in intron 2 of a known schizophrenia-susceptibility gene, glutamate receptor delta 1 subunit (GRID1) [27]. However, expression of miR-346 appears to be driven independently from GRID1 expression, based on miR-346-GRID1 correlation analyses [27, 28]. Although no specific association (risk or protective) has been identified near the GRID1 locus for AD, it may be noteworthy that genetic risks for schizophrenia and AD may be at least somewhat inversely related [30], although specific genes highlighted in the reference are not reported to be regulated by miR-346.

**Fig. 1:**
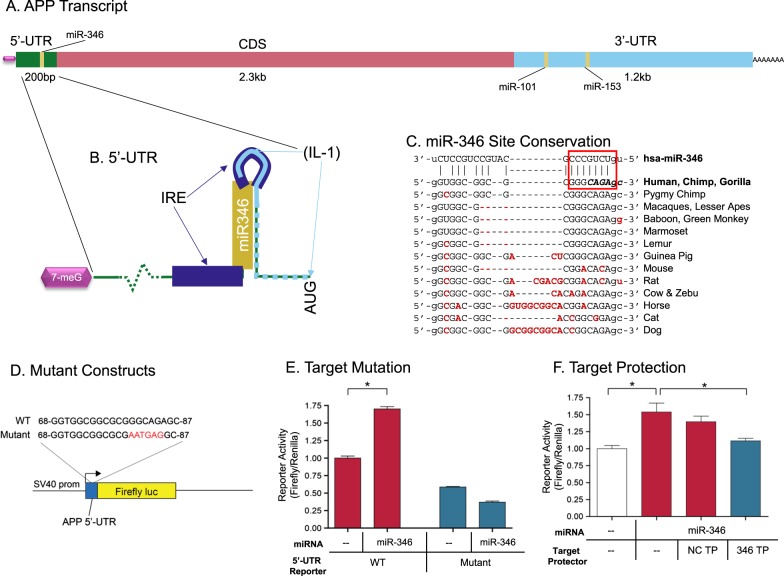
miR-346 targets human APP 5′-UTR via a target site overlapping a known  iron-responsive element (IRE). **a** Schematic of the APP transcript indicating relative sizes of 5′-UTR, coding sequence (CDS), and 3′-UTR. Locations of miR-101, -153, and -346 binding sites also indicated. Binding sites for multiple other miRNAs are omitted for clarity. **b** Diagram indicating miR-346 target site in the 5′-UTR, along with a known IRE and an interleukin-1 acute box (IL-1) that each partially overlap the miR-346 site. The IL-1 acute box reference consensus motif is solid light blue, with the remainder of the APP 5'-UTR fragment that responded to IL-1 treatment indicated with a dashed line. **c** Sequence and predicted base-pairing of human miR-346 with its predicted target site in the human APP 5′-UTR, including the seed sequence interaction (red box). Sequences from multiple mammalian species, orthologous to the predicted miR-346 target site in the human APP 5′-UTR are shown. Red text highlights nucleotide differences of other species' sequences when compared to human sequence. Bold, italicized, black text in human APP 5′-UTR sequence represents fragment of functional IRE consensus sequence. **d** APP 5′-UTR reporter construct containing the APP 5′-UTR sequence inserted upstream of a firefly luciferase CDS. Predicted target site in the 5′-UTR reporter construct was mutated by cassette mutagenesis. Red text highlights mutations introduced in seed sequence. **e** Wildtype and target site mutant reporter luciferase expression. **f** WT 5′-UTR APP reporter construct co-transfected with miR-346 along with either 200 nM negative control target protector or putative miR-346-APP 5′-UTR target protector. *p < 0.05, *n* = 6. “NC TP”: negative control target protector; 346 TP: target protector for APP 5′-UTR recognition site of miR-346

We now demonstrate herein unique characteristics for miR-346. First, unlike most miRNAs, miR-346 interacts with the APP 5′-UTR (Fig. 1a). Second, miR-346 upregulates APP mRNA translation. Third, the specific effect of miR-346 on APP expression is enhanced by intracellular iron chelation with deferroxamine in  human primary neuronal enriched cultures. Finally, this target site for miR-346 overlaps with active sites for iron response protein 1 (IRP1) and an interleukin-1 (IL-1) acute box (Fig. 1b). In addition, this segment of the APP 5'-UTR may respond to other cytokines, including transforming growth factor (TGF)α and TGFβ [89].

We chose to do the bulk of our work in human primary neuronal enriched cultures because our characterization revealed that they showed critical similarities to active neurons in an accompanying matrix of cells that render it particularly valuable for neurological research. These cultures were viable in vitro up to at least 40 days in culture. Cell morphology included neuronal morphology, with a network of processes [31]. Immunocytochemistry revealed the presence of pan-neuronal, astrocytic (GFAP) [31], and neuroprogenitor (nestin-1) (Supplemental Fig. 1) markers, distinct to individual cells. Protein characterization of cultures showed the presence of neuron-specific enolase, GFAP, and synaptosome associated protein-25 [31]. The cultures contain serotonergic, dopaminergic, and GABAergic neuronal cells, although the preponderance of each changes over culture age [31]. Of particular note, these cultures contained cells that were not only morphologically and biochemically neuronal, but cultures also had neuronal functional response, as measured by KCl depolarization [31]. Over time, mature neuronal population within the cultures increased [31]. Finally, the cultures were practical for transfection studies [31]. As such, we deemed them an appropriate model for the present work of exploring neuronal effects of miR-346 upon the APP 5′-UTR.

Based on our present work, we propose a “FeAR” (Fe, APP, RNA) nexus in the APP 5′-UTR that comprises an overlapping miR-346-binding site and the APP IRE. When a “healthy FeAR” exists, activities of miR-346 and IRP/Fe interact to maintain APP homeostasis. Disruption of an element that targets the FeAR nexus would lead to pathogenic disruption of APP translation and protein production.

## Materials and methods

### Prediction of miR-346 binding site in APP 5′-UTR

We scanned the APP 5′-UTR with the miRanda utility on the RegRNA web server [26] to determine potential miRNA recognition sequences.

### Alignment of mammalian APP 5′-UTR sequences

Sequences corresponding to the APP 5′-UTR from 28 species were downloaded from GenBank and aligned with WEBPRank [32]. Total information content of alignments with two major gaps was calculated as ${\sum} {\left( {2 + \mathop {\sum}\nolimits_{b = a}^t {f_{b,i} \times \log _2f_{b,i}} } \right)}$ [33], where *f*_*b*,*i*_ is the relative frequency of a nucleotide (A, C, G, T) *b* at position *i*. SE for information was estimated by $\sqrt {{\sum} {\left( {\frac{1}{{\ln 2}} \times \frac{3}{{2n}}} \right)} ^2}$, where *n* is the number of sequences at a position without a gap.

### Generation of mutant miR-346 site APP 5′-UTR reporter clone

The pGAL reporter construct was used to study regulatory effects on the APP 5′-UTR [13]. Mutagenesis at a predicted miR-346 target site in this 5′-UTR proved refractory to standard site-directed mutagenesis procedures. Therefore, cassette mutagenesis was employed instead. In this form of mutagenesis, the region of plasmid DNA to be mutated was excised by restriction digest. A mutagenized version of this cassette was synthesized, annealed, digested, and ligated into the linearized vector. The oligonucletoides to replace the miR-346 target site in the APP 5′-UTR were obtained from Integrated DNA Technologies (Coralville, IA). We double digested pGAL with *Hin*dIII and *Nco*I. The digested plasmid was resolved by agarose gel electrophoresis, bands containing linearized plasmid were excised and purified with QIAQuick Gel Extraction kit. Mutant oligonucleotides were designed so that, once annealed, the 5′ and 3′ ends would form sticky ends to match *Hin*dIII and *Nco*I sites in the linearized pGAL. Oligonucleotides were annealed and directly ligated into linearized pGAL by combining annealed cassette, linearized pGAL, T4 ligase buffer, and T4 ligase in a 20 µL final volume and incubating at room temperature for 2 h. Approximately, 1 µL of ligase reaction mix was then transformed into Z-competent *Escherichia coli* and plated overnight. True clones were confirmed by direct sequencing of plasmid DNA. Mutagenic oligonucleotides were miR-346mut 5′:

5′-AGCTTAGTTTCCTCGGCAGCGGTAGGCGAGAGCACGCGGAGGAGCGTGCGCGGGGGCCCCGGGAGACGGCGGCGGTGGCGGCGCG**AATGAG**GCAAGGACGCGGCGGATCCCACTCGCACAGCAGCGCACTCGGTGCCCCGCGCAGGGTCGCGC-3 and miR-346mut 3′: 5′-CATGGCGCGACCCTGCGCGGGGCACCGAGTGCGCTGCTGTGCGAGTGGGATCCGCCGCGTCCTTGCCTCATTCGCGCCGCCACCGCCGCCGTCTCCCGGGGCCCCCGCGCACGCTCCTCCGCGTGCTCTCGCCTACCGCTGCCGAGGAAACTA-3′. Boldface indicates specifically mutated nucleotides. Sequencing oligonucleotides was 5′-CTGCTGTGCGAGTGGGAT-3′.

### Cell culturing

HeLa (human cervical adenocarcinoma), U373 (human brain astrocytoma), SK-N-SH (human bone marrow neuroblastoma), 293T (human kidney epithelial), and NT2 (NTera2/D1, human lung carcinoma) cells were obtained from American Tissue Type Culture Collection (ATCC, Manassas, VA). Cells were grown on tissue-culture treated plasticware (Corning, Tewksbury, MA) and maintained at 37 °C in a humidified incubator containing 5% CO_2_. HeLa, 293T, U373, and SK-N-SH were grown in minimum essential media (MEM) (Cellgro, Manassas, VA) supplemented with 10% fetal bovine serum (FBS) (Atlanta Biologicals, Lawrenceville, GA) and 1% penicillin–streptomycin–amphotericin solution (Cellgro). NT2 cells were maintained in Dulbecco’s modified eagle’s media (Cellgro) supplemented with 10% FBS and 1% antibiotic cocktail. Cells were routinely assessed for mycoplasma contamination by use of a commercially available assay. Short tandem repeat profiling was not routinely performed over the course of these experiments.

We cultured human primary neuronal enriched ("human primary") cultures according to procedures we developed and reported [31]. Primary cultures were prepared from the brain parenchyma of aborted fetuses (80–100 days gestational age). The tissues were obtained from the Birth Defects Research Laboratory (BDRL) at the University of Washington with approval from the Indiana University Institutional Review Board (IRB). Fetal brain materials (10–20 g) were shipped overnight in chilled Hibernate-E medium (Invitrogen, Grand Island, NY) supplemented with 1 × B27 serum-free supplement (Invitrogen), 0.5 mM GlutaMAX (Invitrogen), and antibiotic–antimycotic solution (Cellgro).

Tissues were digested in 0.05% trypsin/0.53 mM ethylenediaminetetraacetic (EDTA) acid solution and incubated in a shaking water bath (150 rpm) at 37 °C for 15 min. Trypsin-digested tissues were transferred to Hibernate-E and triturated several times with a siliconized, fire-polished pipette followed by centrifugation at 400×*g*, 15 min. The cell pellet was resuspended in Hibernate-E and triturated once more followed by centrifugation. The pellet was resuspended in culture medium (see below) and cells counted by Trypan blue exclusion.

Cells were plated at a density of 2–4 × 10^5^ cells per well on poly-D-lysine (Sigma-Aldrich, St Louis, MO) coated 24-well plates in Neurobasal medium (Invitrogen), supplemented with 1 × B27, 0.5 mM GlutaMAX, 5 ng/ml Basic fibroblast growth factor (bFGF, Invitrogen) and antibiotic–antimycotic cocktail. Half media changes were performed every fourth day of culture. Cell culture health was assessed by Cell-Titer Glo (CTG) luminescent cell viability assay (Promega, Madison WI), which measures ATP generation.

For those experiments wherein cells were treated with deferroxamine mesylate (DFO, Sigma-Aldrich, St. Louis MO), the appropriate volume of DFO was prepared from a 5 mg/ml stock solution in phosphate-buffered saline (PBS) and added to human primary cell culture plates approximately one hour prior to transfection or HeLa cells were treated with DFO added to cultures for 72 h before harvesting.

### Transfection of DNA vectors or RNA oligonucleotides into cell lines and primary cultures

We transfected several commercially obtained miRNA and siRNA molecules (Supplemental Table 1). During all transfections, antibiotics were omitted from cell culture media. Lipofection was used for all transfections, either with Transfectin (Bio-Rad, Hercules, CA) or Lipofectamine RNAiMAX (Invitrogen). In all experiments where negative control RNA oligonucleotides (i.e., miRNA mimics, miRNA inhibitors, target protectors) were transfected, we used universal negative controls (Supplemental Table 1). These controls are not scrambled sequences and therefore do not necessarily have base composition identical to the experimental oligonucleotides for which they serve as controls.

In those experiments that used the pGAL reporter construct with luciferase-expressing cassette or mutated pGAL, we transfected into HeLa cells. HeLa cells (5 × 10^4^ cells per well) were cultured in white-walled 96-well plates, each well containing 100 µl of serum-supplemented media/well, and transfected with 150–300 ng of reporter constructs using Transfectin. Transfection complexes were prepared by incubating DNA in 20 µl per well of serum-free medium, with 0.75 µl Transfectin per well for 15–20 min. Mixture was directly added to cells on-plate in serum-containing media. Luciferase assays were performed 48 h after transfection.

HeLa cells were co-transfected with reporter constructs and miRIDIAN miRNA mimics (Dharmacon, Lafayette, CO) by incubating HeLa cells cultured in 96-well plates (5 × 10^4^ cells per well) with 150 ng reporter DNA and 40 nM miRNA mimic using 0.2 µl Transfectin per well. Transfection complexes were prepared as described herein.

We did single transfections of Silencer Select siRNA (Applied Biosystems, Carlsbad, CA), miRNA mimics or miRNA target protectors (Qiagen, Valencia, CA) into HeLa or U373 cells using RNAiMAX reagent (human primary cultures  discussed below). For most experiments, HeLa cells (1.35 × 10^5^ cells per well) and U373 cells (7.5 × 10^4^ cells per well) were cultured onto 24-well plates and reverse-transfected [34]. In reverse transfections, transfection complexes are added to cultures at the same time as cells are plated. Cells are initially transfected in suspension until they settle and adhere onto the plate. HeLa cells were transfected with either 20 nM siRNA, 50 nM miRNA mimic, or 100–1000 nM miRNA target protector (TP) using 0.5 µl RNAiMAX per well. Transfection complexes were prepared in 50 µl Opti-MEM serum-free media (Invitrogen) with 10–15 min incubation periods prior to mixing with cell suspensions. U373 cells were similarly transfected with 75 nM miRNA mimics using 3.5 µl RNAiMAX per well. In several cases, miRNA mimics were co-transfected into HeLa cells with siRNA or miRNA target protectors. In these cases, RNAiMAX levels were boosted to 1 µl per well to account for the increase in nucleic acid content.

Multiple batches of human primary cultures were transfected at days in vitro (DIV) 17 in 24-well plates. Cultures were transfected with 20 nM siRNA, 150 nM miRNA mimics, and 1000 nM LNA miRNA inhibitors (Exiqon, Woburn, MA), using 1.25 µl RNAiMAX per well. bFGF supplementation was omitted from media during transfections. In one series of experiments, human primary cultures were transfected with miRNA mimics in the presence of 150 µM DFO. The appropriate volume of DFO was prepared from a 5 mg/ml stock solution in PBS and added to cell culture plates ~1 h prior to transfection.

In all experiments, employing transfection of small RNA oligonucleotides, transfection efficiency was assessed qualitatively by including a siRNA transfection (20 nM) against the gene product of interest. These siRNA were validated in HeLa cells as capable of reducing APP or BACE1 protein and mRNA expression to < 5% of mock or negative control siRNA transfections.

### Human brain samples

Two independent cohorts of brain specimens were utilized in this study. The first set of specimens was provided by Dr. Peter T. Nelson from the University of Kentucky Alzheimer Disease Brain Bank. These specimens were isolated from BA9 of the frontal cortex and consisted of both control (*n* = 5) and AD (*n* = 15) specimens. These specimens were age-matched with a mean age for control specimens of 84.0 ± 2.2 years and 80.8 ± 1.7 years for AD specimens. All AD specimens had advanced AD neuropathology (Braak stage VI and CERAD (Consortium to Establish a Registry for Alzheimer’s Disease) neuropsychological battery score C). CERAD score combines quantification of neuritic plaque in specific brain regions with presence or absence of dementia. Importantly, all specimens were collected following a short PMI (range 1.75–8 h). Finally, the AD component of this cohort consisted of three subgroups defined by history of treatment with AD medications: no history of AD medication (No Rx; *n* = 5), history of treatment with rivastigmine but not memantine (*n* = 5), and history of treatment with memantine but not rivastigmine (*n* = 5).

The second set of specimens originated from the Harvard Tissue Resource Center and was provided by Dr. P. Hemachandra Reddy. These specimens were also isolated from BA9 of the frontal cortex and consisted of control (*n* = 5) and AD (*n* = 15) specimens. Demographic details were previously published [35]. The AD group was further subdivided into three groups defined by stage of neurofibrillary pathology: Braak stage I/II (early AD; *n* = 5), Braak stage III/IV (definite AD; *n* = 5), and Braak stage V/VI (severe AD; *n* = 5). Therefore, this group consisted of specimens spanning the stages of AD progression. Analyses of this cohort was performed either by making comparisons across all Braak stages or by combining control and stage I/II and stage III–VI into two distinct groups for comparison. The rationale for consolidating groups was to increase power of analysis by increasing sample size. Given that stage I/II specimens have only very mild AD pathology and represent a very early-stage of the clinical disease, the assumption is that control and stage I/II specimens are more biochemically similar to one another than to either stage III/IV or V/VI specimens.

Specimens were initially pulverized using a stainless steel chamber, pre-chilled with liquid nitrogen. Pulverized samples were quickly aliquoted and stored at −80 °C, avoiding sample thawing.One aliquot of each sample was processed for protein analysis. This frozen aliquot was immersed in M-PER (ThermoFisher, Waltham, MA) supplemented with 0.1% SDS and protease inhibitor cocktail set III and immediately sonicated using a Sonifier Cell Disruptor 350 (Branson, St Louis, MO) until visible clumps were no longer apparent. Lysates were then incubated with 50 U/mL Benzonase enzyme (EMD, Billerica, MA) for 10 min at 37 °C to reduce nucleic acid content and associated viscosity. Lysates were centrifuged down at 30,000 g for 2 h to clear debris. Cleared supernatants were collected and stored at −80 °C for future protein analysis. For all brain studies, human brain specimens were analyzed in a blinded fashion with diagnostic categories only revealed for data analysis after performing appropriate quality control checks and data normalization. Human brain specimens were provided via external investigators after collection from deceased donors and provided with no identifying information. Therefore, research using these specimens was deemed not to be human subject’s research as defined by HHS and therefore exempt from institutional IRB approval.

### Protein quantification, SDS-PAGE, and western blotting

Cell lysate protein concentrations were measured by bicinchoninic acid assay (Pierce, Rockford, IL) per the manufacturer’s instructions. Protein concentrations were measured with 10 µl of lysate and 200 µl of working reagent at absorbance of 570 nM with a microplate reader (Bio-Rad). All samples were analyzed in duplicates and absorbance values averaged. Concentrations were calculated by comparison to a bovine serum albumin standard curve.

An equal amount of lysate protein (ranging from 1–5 µg) was loaded onto Bis-Tris XT denaturing 10% SDS polyacrylamide gels (Bio-Rad). Proteins were resolved by sodium dodecyl sulphate–polyacrylamide gel electrophoresis (SDS-PAGE) at 200 V for 1.3 h and transferred onto polyvinylidene difluoride by electroblotting at either 30 V for 10 h or 100 V for 1.5 h. Blots were stained with Ponceau-S (Sigma) and visually inspected. Membranes were blocked for 1 h in 5% non-fat milk and incubated overnight with primary antibodies against APP (22C11, Chemicon, Billerica, MA), Dicer (NeuroMAB, Davis, CA), and α-tubulin (B-5-1-2, Sigma-Aldrich). Secondary antibody was HRP-conjugated goat anti-mouse (Rockland Immunochemical, Gilbertsville, PA) or HRP-conjugated rabbit anti-goat (Santa Cruz Biotechnology, Santa Cruz, CA) for 1 h. Bands were visualized using ECL reagent (Pierce, Rockford, IL), detected on film and scanned for densitometric analysis.

### Aβ ELISA analyses

Levels of Aβ40 were measured in the conditioned media (CM) of human primary culture and human brain autopsy samples using a sensitive and specific commercially available ELISA kit (IBL America, Minneapolis, MN). Equal volume of CM (25 µl) was loaded in a plate pre-coated with anti-human Aβ (35–40) antibody (clone 1A10) and incubated overnight. This kit uses HRP-conjugated anti-human Aβ (11–28) as detection antibody. The overall assay was performed according to the manufacturer’s instructions. In brief, CM was added onto pre-coated plates and incubated overnight at 4 °C. The next day plates were vigorously washed with buffer supplied by IBL in the kit and then incubated with detection antibody for ~1 h at 4 °C. Plates were again vigorously washed and then incubated with chromogenic substrate tetramethylbenzidine for 30 mins in the dark. Chromogenic reaction was then stopped by the addition of stop solution and absorbance at 450 nm was read using Tecan GENios microplate reader. Aβ40 values (in pg/ml of CM) were calculated by comparison with an Aβ40 standard curve. This value was normalized to the total lysate protein yield from each well to control for variability attributable to differences in cell number and scaled relative to mock transfection values.

### RT-qPCR analysis of mRNA and miRNA

Both mRNA and miRNA levels were quantified by reverse transcription (RT) quantitative PCR (RT-qPCR). All RT and qPCR steps were performed at a dedicated PCR/RNA workbench with separate supplies to avoid DNA or RNA contamination. For miRNA quantification, stem-loop TaqMan assays were employed (Applied Biosystems, Carlsbad, CA).

Briefly, total RNA (10 ng) was converted to complementary DNA (cDNA) using TaqMan microRNA Reverse Transcription kit (Applied Biosystems) by combining RNA, miRNA-specific RT primer, MultiScribe reverse transcriptase, RNase inhibitor enzyme, dNTPs, reaction buffer and water per the manufacturer’s protocol and incubating reaction mix on a thermocycler at 16 °C for 30 min, 42 °C for 30 min and 85 °C for 5 min. The cDNA was subjected to qPCR using specific TaqMan hydrolysis probe assays (Applied Biosystems). The RT reaction mix (cDNA) was combined with TaqMan miRNA assay and TaqMan Universal PCR master mix (Applied Biosystems) per the manufacturer’s protocol and analyzed on a 7300 Real-Time PCR instrument (Applied Biosystems). Each sample was analyzed in duplicate and signals averaged.

For mRNA quantification, standard mRNA TaqMan hydrolysis probe assays were utilized. Total RNA (10–75 ng) was converted to cDNA with the High Capacity RNA-to-cDNA kit (Applied Biosystems) by combining total RNA, RT enzyme mix, and RT reaction buffer per the manufacturer’s protocol and incubating reaction mix in a thermocycler at 37 °C for 60 min and then 95 °C for 5 min. The RT reaction mix (cDNA) was combined with TaqMan mRNA assay and TaqMan Universal PCR master mix as in miRNA analyses. The PCR reactions were then analyzed on the 7300 Real-Time PCR instrument. Each sample was analyzed in duplicate and signals averaged.

Relative quantification was performed using a modified Δ-Cq method. Relative levels were calculated by taking the ratio of E_x_^ΔCt,x^ for the gene of interest to E_y_^ΔCt,y^ for the stable reference gene, where E_x_ and E_y_ are experimentally determined PCR amplification efficiencies for the gene of interest and reference gene, respectively. This is implemented into the qBase^PLUS^ software used in these studies. In order to determine amplification efficiencies for each TaqMan assay, aliquots of every RNA sample in a given analysis were pooled and used to create a relative standard curve by serial dilution. This standard curve was then converted to cDNA and analyzed by qPCR in parallel with unknown samples. The slope of the plot of Ct versus standard curve dilutions was used to calculate amplification efficiency. For miRNA relative quantification studies, RNU48, RNU49, RNU6B, and miR-16 were used for normalization. For mRNA relative quantification studies, glyceraldehyde 3-phosphate dehydrogenase (GAPDH), β2 microglobulin (B2M), β-actin, and TATA-box binding protein (TBP) were used for normalization.

HPLC-purified synthetic oligoribonucleotide standards were obtained commercially (Sigma and Integrated DNA Technologies), identical in sequence to human miR-101, miR-153, miR-346, miR-339-5p, miR-124, and miR-1. Oligoribonucleotides were resuspended and concentrations measured by A_260_ values. Standard curves with absolute copy counts were prepared by serial dilution, converted to cDNA, and analyzed by qPCR in parallel with unknown samples. Copy counts per reaction were determined from standard curve analysis. Copy counts were then presented as copy counts/15 pg total RNA as a rough estimate of copy counts per average human cell.

### Statistical analyses

Statistical analyses were performed using Prism GraphPad, SPSS, or R using Student’s *t* test and linear or generalized linear models followed by post hoc Dunnett’s *t* test, Šidak-corrected pairwise comparisons, Tukey’s Honest Significant Difference test, or Student–Neuman–Keuls, as appropriate. All tests used *p* ≤ 0.05 as the threshold for significance. Generalized linear models were used whenever data violated the fundamental assumptions of the *t* test or linear models (normality and homoscedasticity). Distribution families and links were chosen by application of the second-order Akaike information criterion [36]. In all cases, error bars represent standard error of the mean. For human brain specimen analysis, the sample size was set to be sufficient to detect a 35% difference in means between groups. Specifically, each brain specimen cohort had 5 control (non-AD) and 15 or 20 disease patient specimens. This sample size was sufficient at power 80% to detect a 32% difference in means with relative standard deviation of 20% for each group, with type I error rate (alpha) of 5%. For cell culture experiments, sample size was determined by multiple previous works in cell culture reasonable sample sizes for our APP and other assays [21–23].

## Results

### Proposed FeAR nexus is well-conserved

Sequence alignment revealed that the FeAR nexus was well-preserved among placental mammals, particularly primates (Fig. 1c, Supplemental Table 2). No homologies were found outside the Eutheria. When expressed in terms of information (bits), primate alignment conservation was 97.44 ± 1.58. Mammalian conservation was 97.72 ± 1.02. This corresponds to a relative information content of 91.9% ± 1.5% and 81.4% ± 0.9% vs. 100% for perfect conservation (106 bits maximum information for primates, 140 for all species). These calculations ignored the two large gaps in the alignment that only had sequences for dog and/or horse. Other gaps were accounted for by an increase in error term value for that position owing to smaller sample size.

### miR-346 activity is through the predicted target site in the APP 5′-UTR

We used the predicted miR-346 target sequence in the APP 5′-UTR (Fig. 1c) to design a mutation (Fig. 1d) in a luciferase expression vector that contained the APP 5′-UTR fused between the SV40 promoter and the firefly luciferase reporter gene [37]. Co-transfection of HeLa cells with the wildtype and mutant luciferase vectors and miR-346 mimic resulted in significant (*p* ≤ 0.05) increase in luciferase signal for wildtype APP 5′-UTR or no alteration by miR-346 for mutated APP 5′-UTR (Fig. 1e). When we co-transfected the wildtype APP 5′-UTR luciferase vector with miR-346 and a TP designed to block the interaction of miR-346 at the predicted APP 5′-UTR target site, we saw a significant reduction of miR-346 mimic effect (Fig. 1f) vs. a negative control TP.

### miR-346 upregulates levels of APP in HeLa cells in a consistent fashion

**Fig. 2:**
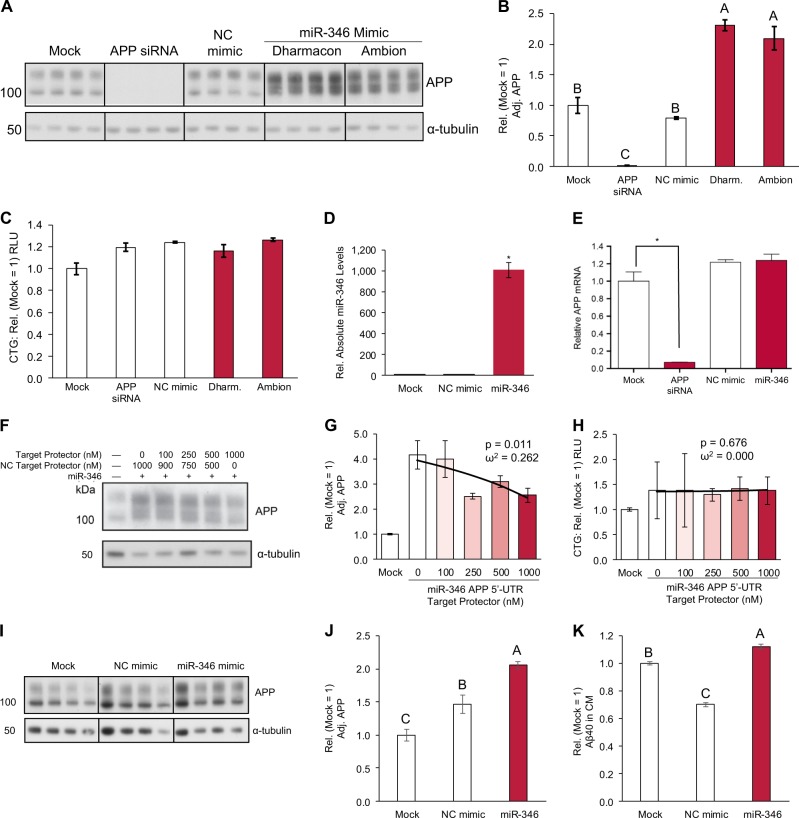
miR-346 delivery dramatically upregulates APP expression in HeLa cells. Induction of APP expression in HeLa by miR-346 delivery is reversed when interaction with the predicted target site in the APP 5′-UTR is blocked**. a** Western blot of HeLa transfected with APP siRNA, negative control miRNA mimic, or miR-346 mimics from two independent sources (Dharmacon or Ambion). **b** APP ( ~ 110–130 kDa by the mAb22C11 probing) signal was normalized to α-tubulin protein ( ~ 51 kDa) signal. APP siRNA significantly (*p* ≤ 0.05) depressed APP, whereas miR-346 mimics both significantly increased it, but each was not different from the other. Letters indicate pairwise statistical comparison (Tukey’s) outcomes. Samples sharing letters are not significantly different. **c** CellTiter-Glo (CTG) cell viability assay of transfected cell cultures. Transfections did not alter overall culture viability (no omnibus or pairwise significant differences). **d** RT-qPCR of miR-346 at 48 h post transfection (two technical replicates), normalized to geometric mean of RNU48, RNU6B and miR16, further scaled relative to mock-transfected levels. RQ = relative quantification; * *p* < 0.05 relative to negative control-transfected cells. **e** APP mRNA RT-qPCR 48 h post transfection (*n* = 3), normalized to geometric mean of β-actin, B2M, GAPDH and TBP, further scaled relative to mock-transfected levels. **f** APP western blot of miR-346 target protection assay with increasing dose of the target protector and fixed amount of miR-346. **g** Blots quantified by densitometric analysis and APP levels normalized to α-tubulin levels, scaled relative to mock transfection (*n* = 4). Linear analysis revealed a significant (*p* = 0.011) dose–response relationship between target protector and reduction of miR-346 activity. **h** CTG of target protector assay cell cultures. No effect appeared from target protector on culture viability. **i** Western blot of miR-346 treatment of U373 cell cultures. **j** Densitometry of APP for U373 cultures was adjusted for α-tubulin. Although NC mimic appeared to increase APP levels, miR-346 induced a greater increase. **k** ELISA of Aβ40 in CM of U373 cells transfected with mock, NC mimic, or miR-346. Transfection with miR-346 significantly (*p* < 0.05) increased levels of Aβ40 in CM

Transfection of HeLa cells with 50 nM of two miR-346 mimics from different commercial sources (Fig. 2a) resulted in elevated (2–2.5-fold) levels of α-tubulin-normalized APP (Fig. 2b). CTG (measuring overall cell culture health) was not perturbed by this treatment (Fig. 2c). We confirmed successful delivery of miR-346 into HeLa cells by RT-qPCR 48 h post transfection (Fig. 2d). Normalized APP mRNA levels in HeLa cells, assayed by RT-qPCR 48 h post transfection, were unchanged (Fig. 2e). RT-qPCR expression levels were normalized to the geometric mean of β-actin, B2M, GAPDH, and TBP expression levels and scaled relative to mock-transfected levels. To confirm binding specificity, we further transfected HeLa cells with miR-346 along with increasing concentrations of a sequence-specific TP. Total transfected nucleic acid concentration was kept constant by adding adjusted amounts of “negative control target protector”. We harvested and lysed cells 72 h post transfection, analyzed protein lysates on SDS-PAGE, and visualized APP and β-actin by western blot on the same membrane (Fig. 2f). We quantified by densitometric analysis and normalized APP levels to α-tubulin levels and scaled relative to mock transfection (*n* = 4).

### Endogenous cellular response to miR-346 is blocked by protection of target site in the APP 5′-UTR

We treated HeLa cells with 15 nM miR-346 mimic and increasing concentrations of TP. We found a significant (*p* = 0.011) inverse relationship between TP dose and miR-346 activity (Fig. 2g). However, CTG-based cell viability was not perturbed (Fig. 2h). The target protection assay established that miR-346 mimics also upregulated endogenous APP mRNA translation. Direct blockade of the specific miR-346 recognition site within the APP mRNA reduced miR-346 mimic activity in a specific dose-dependent fashion.

### miR-346 activity exists in other cell lines

We transfected miR-346 mimics into human glioblastoma U373 cells and analyzed cell extracts on western blot (Fig. 2i). When densitometry was adjusted by corresponding α-tubulin signal, U373 results showed that miR-346 mimic treatment increased APP levels (Fig. 2j). We further evaluated CM for levels of secreted Aβ40 peptide by ELISA and found that miR-346 treatment significantly increased Aβ40 levels in CM of transfected U373 cells (Fig. 2k).

### Activity of miR-346 in the APP 5′-UTR requires the conventional machinery of miRNA activity

**Fig. 3:**
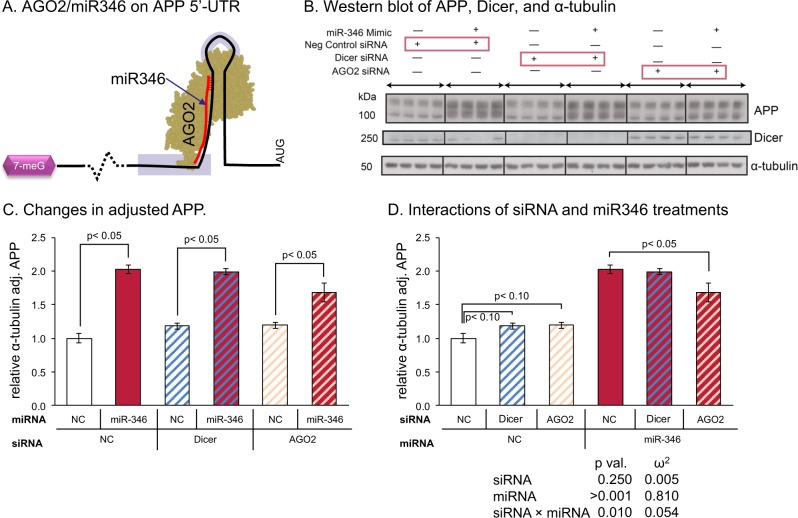
Knockdown of AGO2 but not Dicer attenuates the upregulation effect of miR-346 on APP expression. HeLa cells were co-transfected with or without miR-346 mimic along with either negative control siRNA (NCs), Dicer siRNA or AGO2 siRNA. **a** Model of miR-346 interaction with target APP 5′-UTR sequence. Loading onto AGO2 may provide optimal conditions. **b** APP levels assayed 48 h post transfection by AGO2 or Dicer siRNA by Western blot. **c** AGO2/Dicer siRNA blot (b) as quantified by densitometric analysis, arranged by siRNA treatment. NCs: negative control. In each case, miR-346 co-transfection significantly (*p* < 0.05) increased levels of α-tubulin-adjusted APP densitometry. **d** Quantified AGO2/Dicer siRNA blot outcomes, arranged by miRNA treatment. Knockdown of AGO2 or Dicer did not significantly alter (although *p* < 0.10) adjusted APP signal. However, a difference (*p* < 0.05) in the effect of miR-346 co-transfection occurred when comparing AGO2 knockdown to NCs co-transfected cells

To check the role of the RISC component AGO2 on miR-346 activity (Fig. 3a), we co-transfected HeLa cells with or without miR-346 mimic along with either negative control siRNA, Dicer, or AGO2 siRNA, and measured APP levels of cell lysates 72 h post transfection by western blot (Fig. 3b) followed by densitometry and normalization to α-tubulin levels (Fig. 3c, d). After ANOVA testing interaction of siRNA × miRNA treatments, we found a significant interaction (*p* = 0.010); Šidak-adjusted pairwise comparisons revealed that treatment with siRNA against AGO2 reduced but did not eliminate miR-346 activity (*p* < 0.05).

### Levels of miR-346 and APP diminish as primary human brain cultures mature

**Fig. 4:**
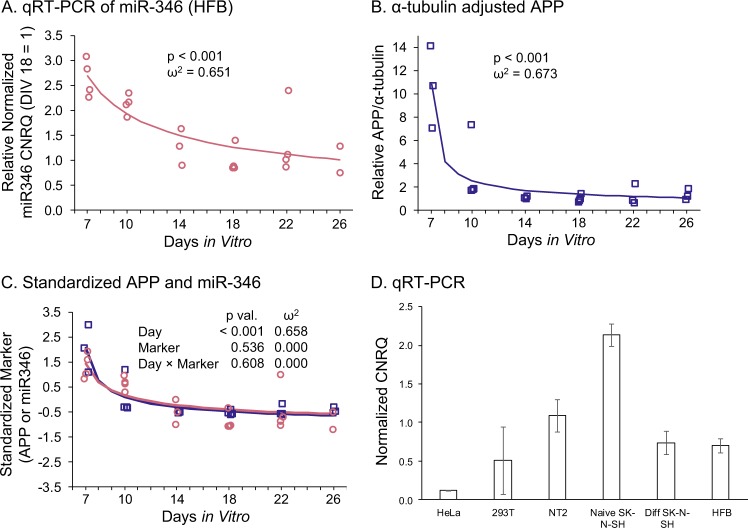
APP and miR-346 levels in human primary culture are in accord with each other over time. Basal levels of miR-346 appear to vary by cell type. **a** RT-qPCR analysis of miR-346 levels across time in human primary culture. RNA expression levels were normalized to the geometric mean of expression levels for endogenous controls RNU6B, RUN48 and miR-16 and then to mean level at day 18 (DIV = 1). These were stated as “CNRQ”, the calibrated normalized relative quantification calculated by qBase^PLUS^. Levels were modeled against DIV. **b** Western blot bands of APP [22] were scanned and densitometrically analyzed, and APP levels normalized to α-tubulin and scaled relative to DIV 18 levels. Levels were modeled against DIV. **c** Combined standardized model. APP and miR-346 signals were standardized (mean subtracted and result divided by standard deviation) and modeled together. Although DIV maintained significance, no significant difference was found between APP and miR-346 standardized levels. **d** Multiple cell lines were cultured and RNA purified as described herein. RT-qPCR analysis of each cell line’s levels of miR-346 was conducted

We cultured human primary cells as described herein and harvested cultures at 7, 10, 14, 18, 22, and 26 DIV. We measured miR-346 levels by RT-qPCR. We determined that miR-346 significantly decreased as cultures aged (Fig. 4a) proportionally to the square of days in culture. We measured APP by western blot [22]. We determined that APP also decreased proportionally to the square of DIV (Fig. 4b). When both miR-346 and APP levels were standardized by subtracting overall means and dividing by standard deviations, the resulting trends were nearly identical (Fig. 4c).

### Native levels of miR-346 apparently differ by cell type

RNA from human cells lines (HeLa, 293T, NT2, SK-N-SH, and SK-N-SH treated with retinoic acid), as well as human primary cultures (18 days in vitro) was prepared and subjected to qRT-PCR for miR-346 levels. Results were normalized to geometric means of corresponding RNU6B, RNU48, and miR16 signals (Fig. 4d). We do not report formal pairwise comparisons, as *N* = 2 for each cell line, although it appears that HeLa had specifically lower levels of miR-346 than other cell lines tested, while human neuroblastoma (SK-N-SH) cells exhibited increased levels of miR-346 in a differentiation specific manner.

### Iron depletion reduces expression of APP in HeLa cultures

**Fig. 5:**
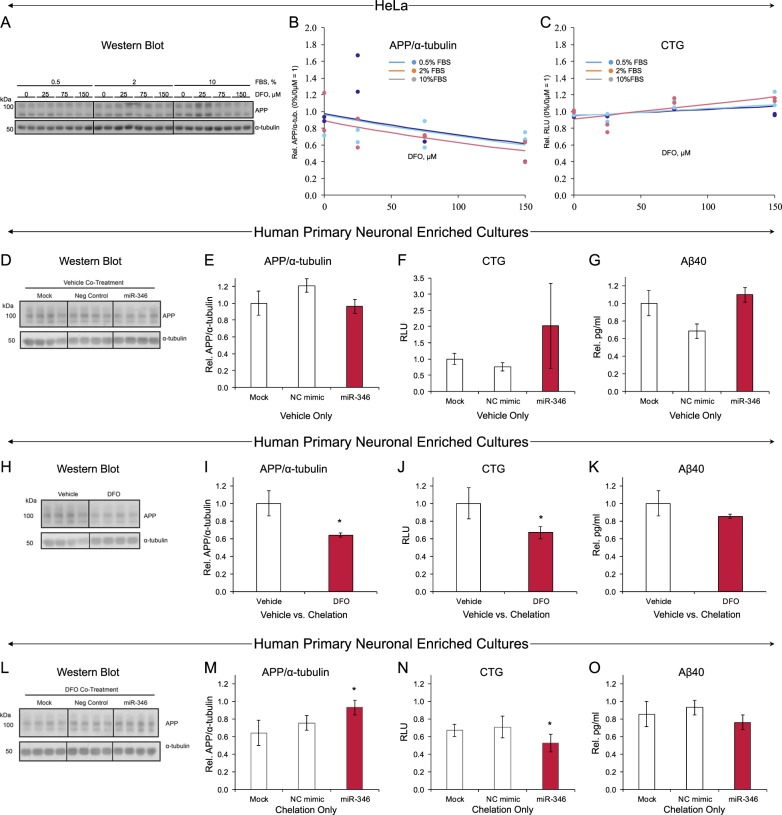
Iron chelation alters APP levels in HeLa cultures and the upregulation effect of miR-346 on APP expression in human primary neuronal enriched culture requires iron chelation. HeLa cultures were treated with different doses of DFO and FBS as described herein. Human primary neuronal enriched cultures were transfected either with negative control mimic or miR-346 mimic, in the presence of vehicle or chelation, and proteins were analyzed on western blots. **a** Western blot of APP and α-tubulin for different treatment combinations of HeLa cells. “Neg Control” is NC mimic. **b** Densitometry (APP/α-tubulin) of HeLa western blot. **c** CTG of treated HeLa. **d** Mock, negative control, or miR-346 treatment of human primary culture without chelation. **e** Densitometry (APP/α-tubulin) of mock/negative/miR-346. **f** CTG of treated cultures. **g** Aβ40 levels in CM of cultures. **h** Vehicle vs. DFO treatment of human primary  culture. **i** Densitometry (APP/α-tubulin) of APP adjusted by α-tubulin of Vehicle vs. DFO. **j** CTG of treated cultures. **k** Aβ40 levels in CM of treated cultures. **l** Co-treatment of human primary culture with DFO and miR-346 (DFO used in all samples). **m** Densitometry (APP/α-tubulin) of DFO co-treatment. **n** CTG of treated cultures. **o** Aβ40 levels of in CM of treated cultures

We treated HeLa cultures with a combination of different concentrations of FBS (0.5%, 2%, 10%) and of DFO (0, 25, 75, 150 µM) and measured APP levels (α-tubulin adjusted) by western blot as well as cell viability by CTG, 72 h after treatment. Two-way modeling of FBS × DFO treatment effects revealed no interaction. However, a significant (*p* ≤ 0.05) effect was found for DFO dose vs. APP and vs. CTG. Notably, DFO reduced APP levels by dose and increased CTG signal (Fig. 5a–c).

### Iron deficiency is necessary for miR-346 effect on APP levels in human primary neuronal enriched cultures

We transfected human primary cultures with miR-346 (Fig. 5d–g); treated them with DFO, 150 µM (Fig. 5h–k); or combined miR-346 transfection and DFO treatment (Fig. 5l–o). We quantified APP levels (adjusted by α-tubulin) by western blot followed by densitometry. In contrast to HeLa culture results, transfection with miR-346 in isolation did not alter APP levels. DFO treatment, alone significantly (*p* ≤ 0.05) reduced adjusted APP. However, when transfected under iron-deficient conditions (DFO chelation), treatment with miR-346 not only reversed chelation effects but increased APP levels beyond untreated culture levels. However, no treatment with DFO or miR-346, alone or combined, significantly altered Aβ40 levels in CM samples. We propose, therefore, that under physiological conditions, miR-346 activity on the APP 5′-UTR depends upon iron deficiency [38].

### miR-346 levels are reduced in AD, particularly in later Braak stages whereas AD increases

**Fig. 6:**
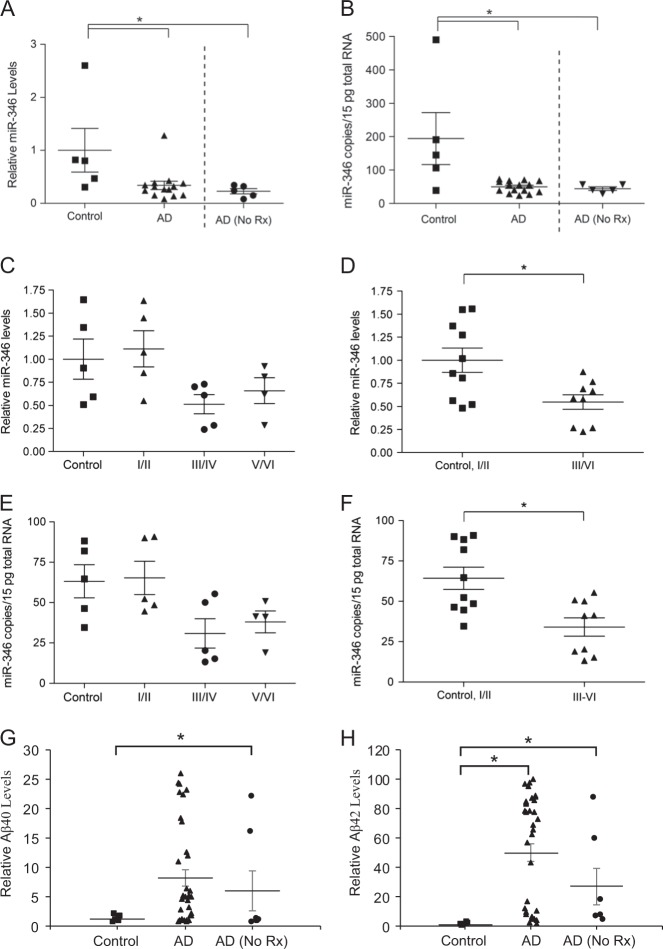
Analysis of miR-346 and Aβ levels in human brain specimens. RT-qPCR analysis of expression levels for miR-346 in brain specimens from AD and control patients in both cohorts. “AD (No Rx)” represents a subgroup of AD patients in cohort 1 that had no history of treatment with cholinesterase inhibitors or memantine. **a**, **c**, **e** expression levels vs. different Braak stages were determined using the modified ΔC_q_ relative quantification method as implemented in qBase^PLUS^ software. Expression levels vs. different Braak stages were normalized to the geometric mean of four endogenous controls: RNU6B, RNU48, RNU49, and miR16. In **b**, **d**, **f**, expression levels vs. different Braak stages were quantified in absolute terms as miRNA copy counts per 15 pg of total RNA. Copy counts were calculated from standard curves prepared from serial dilutions of miRNA oligonucleotide standards with known concentrations. (*p < 0.05). **g** Aβ40 levels in brains of cohort 1 patients. **h** Aβ40 levels in brains of cohort 2 patients

We analyzed two different cohorts of human brain tissue specimens. Both cohorts included individuals with neuropathological AD and age-matched non-AD controls. We performed RT-qPCR analysis of miR-346 levels in brain specimens from AD and control patients in both cohorts (Fig. 6a–f) and Aβ peptides in cohort 1 (Fig. 6g, h). “AD (No Rx)” represents a subgroup of patients from the AD group that had no history of treatment with cholinesterase inhibitors or memantine. We normalized “Relative” expression levels to the geometric mean of four endogenous controls: RNU6B, RNU48, RNU49, and miR-16. We calculated “copies/15 pg total RNA” from standard curves prepared from serial dilutions of miRNA oligonucleotide standards with known concentrations. We normalized levels of Aβ40 and Aβ42 to means of Control samples. In cohort 1 (Fig. 6a, b), both all AD and AD without medications had significantly (*p* ≤ 0.05) lower levels of miR-346 than controls. We found no significant differences if analyzing each Braak stage group in cohort 2 (Fig. 6c–f) separately. When we combined Braak stages as “Control, I/II” vs. III through VI, we found that the reduction in miR-346 according to Braak stage was significant. We likewise found that relative levels of both Aβ peptides were significantly higher in “no Rx” AD samples than controls. Aβ42 levels were also significantly higher than controls for all AD. Although Aβ40 levels were elevated for AD including drug-treated patient samples, the difference was not significant (Fig. 6g–h).

## Discussion

APP plays a central role in AD etiology and progression. In this report, we address novel features of regulation by miRNA of APP mRNA translation. Among its many functions, APP has metal-associated redox activity [12] and stabilizes the plasma membrane for Fe transport (with or without ferroxidase activity) [11, 39]. Thus, preventing disruption of Fe metabolism is a worthwhile target of AD research [40].

Several miRNAs, including miR-101, miR-153, and miR-298, regulate APP mRNA translation [21, 22, 41]. To discover further miRNA regulators of APP, we scanned the APP 5′- and 3′-UTRs with the miRanda utility in the RegRNA online database [26] and found a putative target site for miR-346 in the 5′-UTR. When tested, miR-346 strongly upregulated expression of an APP 5′-UTR reporter clone and endogenous APP protein in HeLa cells. Site mutagenesis and TP transfections demonstrated that these effects were mediated by specific interaction with the predicted APP 5′-UTR target site (Figs. 1–2). We also observed an upregulatory effect in human primary cultures but only after iron chelation. Therefore, miR-346 has “non-canonical” (stimulative/disinhibitive) regulatory effects on APP expression via a “non-canonical” target site in the APP 5′-UTR that likewise contains an IRE. Inhibiting the interaction we observed may be a viable therapeutic strategy for potentially regulating APP expression and Aβ production in the AD brain.

Early exploration into the upregulation of mRNA translation by miRNAs concentrated on conventional 3′-UTR region targets. In those cases, it was determined that miRNAs would direct AGO and fragile-X mental retardation syndrome-related protein (FXR1) toward AU-rich areas (ARE) of the 3′-UTR, and many miRNA target sites (~ 75%) are within AREs. Furthermore, this effect can switch from stimulation to repression depending on whether cells are quiescent or dividing [42]. FXR1 is a homolog of FMRP, which is known to repress translation of APP [43]. However, more recent work has determined that stimulation of translation by miRNA is not limited to targeting the 3′-UTR, nor is it limited to interactions with ARE [44]. Instead, multiple pathways can operate that involve either the 3′- or 5′-UTR and several potential protein partners, although AGO2 is usually (but not always) present [44]. It bears noting that the miR-346 site in the APP 5′-UTR is *not* within an ARE. What is a particularly interesting contrast is that our own work demonstrated that miR-346 stimulation at least partially required AGO2, while miR-346 stimulation of RIP140 was enhanced by knockdown of AGO2 [45]. Thus, specific action of a particular miRNA on a specific mRNA may depend closely upon local metabolic conditions.

While most known miRNA regulatory interactions are limited to the mRNA 3′-UTR [46], several examples exist of effective miRNA targeting in the 5′-UTR or CDS [45, 47], and some even target both 5′- and 3′-UTRs in a single mRNA [48]. The vast majority of validated miRNA:mRNA target interactions inhibit target translation. Nevertheless, several examples exist of apparent stimulation by miRNA on target expression [44]. Most specifically, our results for APP are similar to miR-346 regulation of receptor-interacting protein 140 (RIP140) [45]. That is, miR-346 stimulates translation through the RIP140 5′-UTR.

Specific differences have been reported between miRNA activities in quiescent (G0) vs. actively dividing cells [42]. Given that we have evidence that our human primary culture contains a significant portion of mature neurons [31], we believe that our results adequately reflect one such difference, particularly since the effects of miR-346 in HeLa (immortalized, actively dividing cells) were not identical to those we observed in human primary cultures.

A potentially pertinent pathway for AD is miR-346 regulation of the unfolded protein response (UPR) [29]. This pathway activates under accumulation of unfolded proteins in the ER. Activation of UPR results in the inhibition of global protein production and targeted induction of gene expression for products that increase ER protein folding capacity [49]. Expression of miR-346 increases UPR through UPR-linked transcription factor XBP1 [29]. This leads to decreased expression of TAP1 through interaction between miR-346 and the *TAP1* 3′-UTR. TAP1 is an ATP-binding cassette transporter that translocates antigens derived from proteasomal processing into the ER lumen for loading onto MHC antigen receptors. Notably, miR-346 also decreases MHC class I gene expression via indirect interactions, further implicating miR-346 as an immunomodulatory miRNA.

To bring this into context with our present work, neurons in the AD brain are often invested with NFT consisting of aggregated hyperphosphorylated tau protein that might be expected to induce ER stress. UPR is activated in pretangle neurons [50]. Given that UPR is active in the AD brain and that APP expression is elevated following UPR activation [51], it is reasonable to speculate that miR-346 expression may also be induced in certain cells of the AD brain and drive APP expression in pretangle neurons. Even broader associations between neurodegeneration and UPR likely exist [52]. Multiple neurodegenerative diseases, including AD, Parkinson’s disease, Huntington’s disease, and amyotrophic lateral sclerosis have association with activated UPR [53]. In AD, a specific UPR-related mechanism may be autophagy [54]. Furthermore, UPR may contribute to AD amyloidosis. Specifically, X-box binding protein 1 (XBP1) is a transcription factor that regulates ADAM10 [55]. ADAM10 is the primary α-secretase, which drives APP processing away from amyloidogenic Aβ production. XBP1 is differentially spliced during UPR [56]. This specific splicing difference likewise alters XBP1 activity on ADAM10. In brains from AD, normal XBP1 and ADAM10 mRNA levels were below those of non-AD controls [55]. Of particular pertinence, Fe depletion reduces the ability of cells to mount UPR against ER stress, and this is relieved by Fe supplementation [57].

Mechanisms involved in post-transcriptional miRNA-mediated inhibitory regulation are fairly universal and well described [58]: AGO2 as a member of RISC recruits GW182 to the target transcript, promoting further protein interactions that lead to translational inhibition and transcript deadenylation and degradation [58, 59]. To explore the mechanism underlying the upregulation effect of miR-346 on APP mRNA translation, we tested involvement of proteins implicated in canonical miRNA biogenesis (Dicer) and function (AGO2). Upregulation of APP by miR-346 was significantly reduced when expression of AGO2 was knocked down. AGO2 was originally discovered as a component of a molecular complex involved in translation initiation [60]. This function has since gone largely unexplored. Given the location of the miR-346 target site in the APP 5′-UTR, near the site of ribosome assembly, one possible explanation for the requirement of AGO2 is that it may mediate the upregulation effect via its function in translation initiation. Another possibility is that AGO2 may be required to sterically inhibit interactions between inhibiting trans-factors and the APP 5′-UTR IRE.

The miR-346 target site in the APP 5′-UTR directly overlaps with a known IRE and an IL-1 acute box [13, 14]. The IRE located within the APP 5′-UTR binds IRP1 but not IRP2 [13, 14, 19]. It is possible that miR-346 activity may in some way interact with IRP1 and/or IL-1 activity through their co-localized target sites on the APP 5′-UTR, particularly IRP1. In this regard, IRP1 inhibits APP translation when bound to the 5′-UTR IRE. When iron levels are increased, IRP1 binds free iron and dissociates from the APP mRNA allowing translation to proceed uninhibited. When iron levels are decreased (such as with iron chelation), free iron dissociates from IRP1 allowing IRP1 to bind to the APP 5′-UTR IRE and inhibit APP translation. IL-1 participates in Fe homeostasis indirectly, through inflammatory cascades. In particular, IL-1 increases recruitment of IRP1 by transient increase of the labile Fe pool [61]. Further, IL-1 stimulates translation of APP mRNA through its 5′-UTR [62]. In primary human primary cultures, miR-346 activity was absent unless Fe levels were reduced by chelation with DFO. Although it is tempting to speculate that the potent effect of miR-346 on APP levels in HeLa cells could be attributed to relative Fe deficiency, we have no direct evidence of this as we did not measure free Fe levels in media. In fact, media supplementation with FBS would be expected to provide Fe both in free form and bound to transferrin. Further, it is not clear that comparing media iron levels would reflect differences in intracellular free iron levels. Therefore, the exact mechanism whereby miR-346 regulates APP levels in HeLa cells requires further investigation.

Nevertheless, our work allows us to build an extended model of miR-346’s role in APP’s promotion of export of Fe from the cytosol to the extracellular space. Aside from its role in regulating APP expression, Fe, along with Cu and Zn, bind to Aβ, particularly in plaque cores [63], and slows the normal ordered progression of Aβ to higher ordered aggregates, such as fibrils. This Fe interference promotes Aβ toxicity in neuronal cells [64]. Fe bound to Aβ also accelerates ROS formation [65]. Thus, therapies that modulate Fe homeostasis in the AD brain have been proposed as a means of reducing Aβ-associated Fe toxicity and reducing APP translation and Aβ production [20, 66, 67]. This may be a chicken-or-egg question: does Fe accumulation, exacerbated by perturbation of miR-346-dependent regulation of APP, lead to AD, or does it merely exacerbate symptoms after the disease already exists?

In addition to Fe, several other metals play some role in the production of APP and Aβ. These include lead (Pb) [39], copper (Cu) [68–70], and manganese (Mn) [71, 72, 87]. Their contributions are complex and often not overlapping. Cu, in particular, appears to regulate transcription and translation [69]. However, it may be a complex relationship. Although Cu supplementation stimulated APP 5′-UTR activity [70], net effects may vary by tissue [68–70]. It is noteworthy that Cu binds IRP1 and reduces its ability to bind mRNA, although at less efficiency than Fe [73]. Pb enhances IRP1 inhibition of APP translation via enhancing IRP1:APP 5′-UTR interaction [39]. Shorter-term exposures to Pb also increases IRP1 levels before resulting in lower levels with more extended exposure. This operates through Pb disruption of extracellular signal-regulated kinase 1/2 [74]. A neurotoxic effect has also recently been explicitly measured for Mn, via suppression of APP 5′-UTR activity [87].

In our studies, miR-346 upregulated Aβ in U373 human astrocytoma cells but did not have a significant effect on Aβ levels in primary human cell cultures. In AD brain samples, miR-346 was significantly downregulated in late-Braak stages. We had previously reported that both miR-101 and miR-153 were also downregulated in late-Braak AD, accompanied by significant elevation of Aβ and APP [21, 22]. If miR-346 is to upregulate APP, why would it be deficient in AD brain? We admit that late-stage reduction in miRNA species, without early-stage or prodromal evidence, could reflect a general breakdown in miRNA regulation that cuts across specific functions or be an epiphenomal change reflecting broad changes in the relative number of different cell types as neurodegeneration progresses. If dysregulation of APP’s contribution to Fe homeostasis plays a role in AD, that role would be in earlier stages of the disorder, such as mild cognitive impairment (MCI) and Braak stage I, and may not be reflected in the “accumulative phase” (Braak II +).

**Fig. 7:**
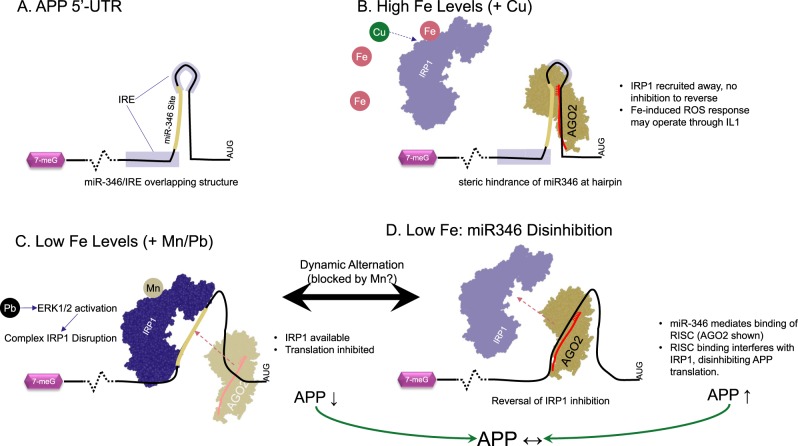
FeAR nexus model of miR-346 participation in Fe homeostasis and AD risk. Schematic illustrates interaction of miR-346 and IRP1 at “FeAR nexus” in APP 5′-UTR. **a** Structure of the APP 5′-UTR illustrating a putative hairpin loop [13, 15, 19] that includes both the IRP1 site and miR-346 recognition sequence. **b** During Fe influx, IRP1 is recruited away from the APP 5′-UTR, no longer inhibiting APP translation. Although this may “free” the APP 5′-UTR to bind with miR-346/RISC (RISC represented by Ago2), the apparent stimulative activity is parsimonously explained by disinhibition vs. IRP1. When IRP1 is not inhibiting, binding by RISC offers no additional stimulative effect. Cu also has some activity in recruiting IRP1 away from the APP 5'-UTR. Cu has a lower affinity for IRP1 but is still able to bind and partially recruit it away. **c** If Fe levels are low, IRP1 is not recruited away and binds the APP 5′-UTR, inhibiting APP translation. In addition, Mn may bind to IRP1 and prevent its recruitment by Fe or otherwise interfere in Fe recruitment. Pb activates ERK1/2, which has a complex cascade of consequences, some of which include complex disruption of IRP1 levels. **d** Binding of the miR-346/RISC complex would then disinhibit by displacing IRP1. Alternation of IRP1 inhibition and miR-346/RISC disinhibition would facilitate APP homeostasis

We propose a “first-order” model that incorporates Fe and miR-346, along with “supporting roles” played by Cu and Mn (Fig. 7). Although Zn can bind IRP1 [88], and it blocks APP ferroxidase activity it does not alter APP levels [12]. Under our model, “healthy FeAR” is homeostatic. The IRE and miR-346 sites partially overlap in the APP 5′-UTR (Fig. 7a). Fe influx recruits IRP1 away from the APP 5′-UTR, which may “free” the site, and is equivalent to simple disinhibition (Fig. 7b). When Fe is reduced, IRP1 becomes available and binds its site, inhibiting APP translation (Fig. 7c). Binding of miR-346/RISC would displace IRP1, disinhibiting APP translation in the same fashion that Fe recruitment of IRP1 would (Fig. 7d). This process would alternate back and forth between inhibition and disinhibition, permitting sufficient APP to be translated for its multiple functions [75–78]. Notably, this phenomenon would require the interaction of IRP1 with the 5'-UTR IRE and, therefore, would be expected to be blunted in a setting of “iron excess”, thereby providing a plausible hypothesis for why the stimulatory effect was observed in human primary neuronal enriched cultures only after iron chelation. Further experimental work will be necessary to  better integrate Cu, Mn, Pb, and IL-1 into the Fe-miR-346 activity network. Considering clinical complications, unmodified iron chelation therapy in AD is likely to be a poor treatment strategy. Metal-complexing agents exist with more targeted and less-systemic effects on metal ion binding and redistribution, the so-called metal-protein attenuating compounds [79, 80]. “XH1” binds Aβ and chelates metals. It reduces APP protein expression in neuronal cells [81]. However, idiopathic anemia is a common comorbidity with AD [82], and low hemoglobin associates with greater risk of death among AD patients [83]. On the other hand, in a Japanese study confined solely to dementia patients, subjects had a direct association between greater levels of circulating hemoglobin and brain accumulation of Aβ [84].

In the context of translational implications, we observed that miR-346 levels are reduced in later stages of AD, but we cannot necessarily infer from this that prodromal AD or earlier stages of development are necessarily due to deficiency of miR-346. Several cases of reduced miRNAs have been found in association with AD staging, in particular, we have reported that miR-101 and miR-153, both of which downregulate APP expression, are likewise reduced in AD [21, 22]. This may reflect underlying etiology, or it may reflect general neurodegeneration and glial invasion. If these miRNAs are critical to normal brain function, and they are likewise specifically expressed in neuronal cells, their loss in brain samples may just as well reflect a change in proportion of neuronal vs. non-neuronal cells in diseased brains. Finally, we wish to make note of possible roles for Fe deficiency in another pervasive brain disorder, schizophrenia. Hippocampal Fe deficiency, both with and without systemic anemia, resulted in impaired prepulse inhibition (PPI) of the acoustic startle reflex. Impaired PPI is a reliable measure of the schizophrenia endophenotype of defective sensorimotor gating [85]. While no APP-Fe-Schizophrenia axis has been found, that APP activity includes significant regulation of Fe homeostasis suggests that the miR-346/IRP-1/Fe pathway may function in other neurological disorders.

From an AD etiology standpoint, Fe influx could be part of a cascade of cellular stresses (e.g., redox stress and inflammation) that would initially upregulate miR-346 and, thereby, APP. In healthy conditions, this would eventually result in negative feedback that reduces miR-346 and APP to pre-stress levels. Under pathogenic conditions, negative feedback to miR-346 might be insufficient to halt an APP pathogenic cascade. Other mechanisms would drive excess APP and Aβ, but miR-346 would have “fallen by the wayside”, downregulated as a result of late AD neurobiology. Only further experimental investigation could accurately define the relationships. For example, our future work would consider stimulation of the FeAR nexus by IL-1 [62], and show direct evidence of IRP1-miR-346 competition or how metallic ions other than Fe, such as Cu or Mn, could alter the system. In addition, our future work would use the full UTR sequence, which could add another layer of complexity. It is noteworthy that the 5′-UTR for APP is also transcriptionally active, as we have previously shown [37]. This includes a “CAGA box” that takes part in transforming growth factor β activity in regulating APP transcription [86]. It might be overly simplistic to presume that transcriptional regulation directly interacts with translational regulation merely because both stages happen to have overlapping target regions on the DNA and corresponding RNA sequences. Nevertheless, the presence of such an overlap could open up opportunities for drug modulation that could target both stages through one site.

## Electronic supplementary material

miR346 Supp Table 1

miR346 Supp Table 2

Supplemental Figure S1

Legend to Supplemental Figure S1
