# Supplementary material for: Novel upregulation of amyloid-β precursor protein (APP) by microRNA-346 via targeting of APP mRNA 5′-untranslated region: Implications in Alzheimer’s disease
Source: Mol Psychiatry. 2018 Nov 23;24(3):345–63. doi: 10.1038/s41380-018-0266-3 (PMC6514885; doi:10.1038/s41380-018-0266-3)
Supplement: Supplementary file 1 — miR346 Supp Table 1 [file 41380_2018_266_MOESM1_ESM.docx]

Supplemental Table 1. Transfected siRNA and miRNA molecules

| ***Type*** | ***Name*** | ***Vendor*** | ***ID/Catalog #*** |
| --- | --- | --- | --- |
| siRNA | APP | Applied Biosystems | s1500 |
| siRNA | Negative Control (NCs) | Applied Biosystems | 4390843 |
| miRNA Mimic | miR-346 | Dharmacon | C-300712-03 |
| miRNA Mimic | miR-346 | Ambion | 4464066-MC10238 |
| miRNA Mimic | Negative Control (NCm) | Dharmacon | CN-002000-01 |
| miRNA Target Protector | miR-346 APP Target Site | Qiagen | custom |
| miRNA Target Protector | Negative Control Target Protector | Qiagen | custom |
