# Supplementary material for: Novel upregulation of amyloid-β precursor protein (APP) by microRNA-346 via targeting of APP mRNA 5′-untranslated region: Implications in Alzheimer’s disease
Source: Mol Psychiatry. 2018 Nov 23;24(3):345–63. doi: 10.1038/s41380-018-0266-3 (PMC6514885; doi:10.1038/s41380-018-0266-3)
Supplement: Supplementary file 2 — miR346 Supp Table 2 [file 41380_2018_266_MOESM2_ESM.docx]

Supplemental Table 2. Alignment of “FeAR Nexus” sequences from multiple mammalian species.

| **Species** | **Sequence** |
| --- | --- |
| Hsa-miR-346 | 3’-uCUCCGUCCGUAC------GCCCGUCUgu-5’ |
|  | \| \|\|\| \|\|\| \|------\|\|\|\|\|\|\|\| |
| *H. sapiens* | 5’-CCC-CGGGA-------GACGGCGG------CGGUGGC-GGC--G------CGGGCAGAGCAAGGACGCGGCGGAUC-3’ |
| *Pa. trogoldytes* | 5’-CCC-CGGGA-------GACGGCGG------CGGUGGC-GGC--G------CGGGCAGAGCAAGGACGCGGCGGAUU-3’ |
| *Pa. paniscus* | 5’-CCC-CGGGA-------GACGGCGG------CGGCGGC-GGC--G------CGGGCAGAGCAAGGACGCGGCGGAUC-3’ |
| *G. gorilla* | 5’-CCC-CGGGA-------GACGGCGG------CGGUGGC-GGC--G------CGGGCAGAGCAAGGGCGCGGCGGAUC-3’ |
| *Po. Pygmaeus* | 5’-CCC-CCAGA-------GACGGCGG------CGGUGGC-G-----------CGGGCAGAGCAAGGACGCGGCGGACC-3’ |
| *N. leucogenys* | 5’-CCC-CCGGA-------GUCGGCGG------CGGUGGC-G-----------CGGGCAGAGCAAGGACGCGGCGGAUC-3’ |
| *Macaca^b^* | 5’-CCC-CCGGA-------GACGGCGG------CGGUGGC-G-----------CGGGCAGAGCAAGGACGCGGCGGAUC-3’ |
| *Pa. anubis* | 5’-CCC-CCGGA-------GACGGCGG------CGGUGGC-G-----------CGGGCAGAGGAAGGACGCGGCGGAUC-3’ |
| *Ch. Sabaeus* | 5’-CUC-CCGGA-------GACGGCGG------CGGUGGC-G-----------CGGGCAGAGGAAGGACGCGGCGGAUC-3’ |
| *Cal. jacchus* | 5’-CCC-CCGGA-------GACGGCGG------CGGUGGC-G-----------CGGGCAGAGCAAGGGCGCGGCGGUUC-3’ |
| *Microc. Murinus* | 5’-GCC-CCCGA-------GACAGCGG------CGGCGGC-G-----------CGGGCAGAGCAAGGGCGCGGCGGACC-3’ |
| *T. belangeri* | 5’-CCA-CCGGA-------GAGAGCGG------CGGCGGC-G-----------CGG-CAGAUCAAGGGCGCGGCGGAUC-3’ |
| *Mus musculus* | 5’-GCCACCGGA-------GACGGCGG------CGGCGGC-G-----------CGGACACAGCCAGGGCGCGGCGGAUC-3’ |
| *Mus caroli* | 5’-GCCACCGGA-------GACGGCGG------CGACGGC-GGC--GGCGACGCGGACACAGCCAGGGCGCGGCGGAUC-3’ |
| *Mus pahari* | 5’-GCCACCGGA-------GACGGCGG------CGGCGG---------------GGAUACAGCCAGGGCGCGGCGGAUC-3’ |
| *R. norvegicus* | 5’-GCCACCGGA-------GACGGCGG------CGGCGGC-GGC--GACGACGCGGACACAGUCAGGGUGCGGCGGAUC-3’ |
| *Microt. ochragaster* | 5’-GCUCUCGGA-------GACGGCGG------CGGCG------------ACGCGGACCCAGCCAGGGCGCGGCGGAUC-3’ |
| *Cav. Porcellus* | 5’-ACC-CCGGA-------GACGGCAG------UGGCGGC-GGC--G---ACUCGGGCAGAGCAAGGGCGCGGCGGAUC-3’ |
| *O. cuniculus* | 5’-CCC-CUGGA-------GACGGCGG------CGGCGGC-G-----------CGGGCAGAGCAAGGGCGCGGCGGAUC-3’ |
| *S. scrofa* | 5’-CCA-CCGUA-------GACAGCGG------CAGCG------------ACACCGGCAGAGCAAGGGCGCGGCGGAUC-3’ |
| *Caprinae^c^* | 5’-CCA-CCGGA-------GACGGCGG------CGGCGGC-G-----GCGACACAGGCAGAGCACGGGCGCGGCGGAUC-3’ |
| *Bos^d^* | 5’-CCA-CCGGA-------GACGGCGG------CGGCGGC-G-----GCGACACAGACAGAGCAAGGGCGCGGCGGAUC-3’ |
| *F. cattus* | 5’-CCA-UCGGA----------GACGG------CGGCGGC-G-----ACGGCACCGGCGGAGCCAGGGCGCGGCGGAUC-3’ |
| *C. lupus familiaris* | 5’-CCC-CGAGACGGCGGCGGCGGCGGCGGCGGCGGCGGC-G-----GCGGCACCGGCAGAGCCAGGGCGCGGCGGACC-3’ |
| *E. caballus* | 5’-CCA-CCAGA-------GGCGGCGGCGGCGACGGCGGU-G-----GCGGCACGGACAGAGCAAGGGCGCGGCGGAUC-3’ |
| Primate Consensus^e^ | 5’-CCC CcgGA GACGGCGG CGGuGGC G CGGGCAGAGcAAGGaCGCGGCGGAuC-3’ |
| Consensus^f^ | 5’-cCc CcgGA GaCgGCGG CGGcGGc g CgGgCAgAGcaAGGgCGCGGCGGAuC-3’ |

^a^Portion of the alignment corresponding to the IRE/miR-346/IL1 acute box is shown. Sequences of IRE are underlined. Sequence of IL1 acute box is in blue. Nucleotides that differ from human sequence are red. Homology of all three elements is well preserved among primates. No homology at all was found for non-placental mammals.

^b^Sequence is identical for both *M. mulatta* and *M. fascicularis*.

^c^Sequence was identical for both *Capra hircus* and *Ovis aries*.

^d^Sequence was identical for both *B. taurus* and *B. indicus*.

^e^Capital letters indicate appearance in ≥10 sequences, lower-case letters indicate 6-9 sequences.

^f^Capital letters indicate appearance in ≥25 sequences, lower-case letters indicate 14-24 sequences.
