# Supplementary figures and images for: Novel upregulation of amyloid-β precursor protein (APP) by microRNA-346 via targeting of APP mRNA 5′-untranslated region: Implications in Alzheimer’s disease
Source: Mol Psychiatry. 2018 Nov 23;24(3):345–63. doi: 10.1038/s41380-018-0266-3 (PMC6514885; doi:10.1038/s41380-018-0266-3)

## Slide 1
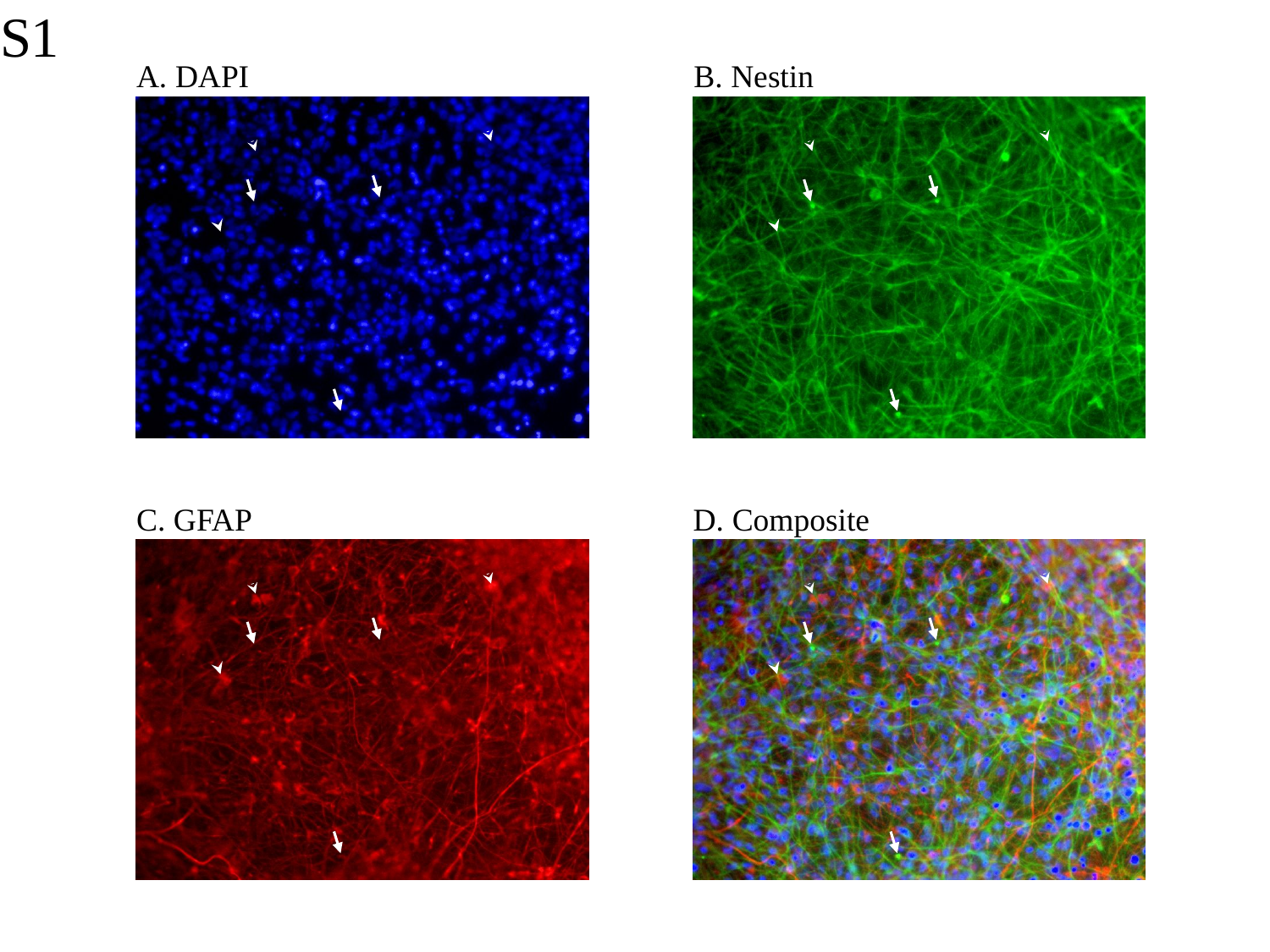

S1
A. DAPI
B. Nestin
C. GFAP
D. Composite

Supplement: Supplementary file 3 — Supplemental Figure S1 [file 41380_2018_266_MOESM3_ESM.pptx]
