# Supplementary material for: Novel upregulation of amyloid-β precursor protein (APP) by microRNA-346 via targeting of APP mRNA 5′-untranslated region: Implications in Alzheimer’s disease
Source: Mol Psychiatry. 2018 Nov 23;24(3):345–63. doi: 10.1038/s41380-018-0266-3 (PMC6514885; doi:10.1038/s41380-018-0266-3)
Supplement: Supplementary file 4 — Legend to Supplemental Figure S1 [file 41380_2018_266_MOESM4_ESM.docx]

**Legend to Supplemental Figure S1. Immunocytochemical characteristics of HFB cultures.** Cells were fixed using paraformaldehyde (4%) and permeabilized by 0.13% triton X-100, followed by blocking with 10% horse serum per standard ICC protocols. Fixed cells were incubated overnight with neuron and glial-specific primary antibodies, specifically mouse monoclonal neuronal progenitor (Nestin, Sigma, 1:500) and rabbit polyclonal anti-GFAP (Sigma; 1:100 dilution). Appropriate secondary antibodies and fluorophores were utilized to obtain fluorescence images. For nuclear staining, a drop of 4’, 6-diamidino-2-phenylindole (DAPI, Sigma, diluted 1.5μg/ml in water) was used. A) DAPI emitted blue flourescence. B) Nestin in green. C) GFAP in red. D) Composite overlay. Arrows show nestin-positive cells that did not have GFAP signal, Arrow-heads show GFAP-positive cells that did not have nestin signal. Figure shows phenotypic character of the culture and also indicates that maturation of neurons (i.e. neuronal precursor and glial markers) do not overlap at DIV 24.
